# Building subnational capacities in animal health to deliver frontline cross-sectoral health services in Kenya

**DOI:** 10.3389/fvets.2023.1150557

**Published:** 2023-08-04

**Authors:** Rinah Sitawa, Evans Tenge, Khadija Chepkorir, Mark Nanyingi, Sam Okuthe, Caryl Lockhart, Harry Oyas, Obadiah Njagi, Mary Teresa Agutu, Jack Omolo, Tequiero Okumu, Charles Bebay, Folorunso O. Fasina

**Affiliations:** ^1^Emergency Center for Transboundary Animal Diseases (ECTAD), Food and Agriculture Organization of the United Nations (FAO), United Nations Office in Nairobi (UNON), Nairobi, Kenya; ^2^Directorate of Veterinary Services, Ministry of Agriculture and Livestock Development, Nairobi, Kenya; ^3^Department of One Health, Institute of Infection and Global Health, University of Liverpool, Liverpool, United Kingdom; ^4^Emergency Center for Transboundary Animal Diseases (ECTAD), Regional Office for Eastern Africa, Food and Agriculture Organization of the United Nations (FAO), United Nations Office in Nairobi (UNON), Nairobi, Kenya; ^5^Food and Agriculture Organization of the United Nations (FAO), Rome, Italy; ^6^Kenya Veterinary Board, Nairobi, Kenya; ^7^Department of Agriculture, Livestock Development and Fisheries, Kilifi, Kenya; ^8^University of Nairobi College of Agriculture and Veterinary Sciences, Nairobi, Kenya

**Keywords:** cross-sectoral engagement, capacity building, ISAVET, localization, Kenya

## Abstract

**Introduction:**

Operationalizing effective subnational veterinary services as major contributor to disease surveillance, reporting, diagnoses and One Health requires resources and mindset change. Here we describe workforce capacity building in animal health in Kenya and an approach that can be used to skill-up this workforce to respond beyond animal health challenges to emergent One Health realities and public health emergencies. Furthermore, triggering a paradigm shift has been identified for impactful delivery of health services, thus mindset change are important for learning new skills, but they also affect the way that we think about everything, for instance training in field epidemiology. Emphasis was therefore placed on skills, beliefs, and mindset shift.

**Methods:**

Contextualized within the Kenyan environment, this description identifies problems likely to be found elsewhere: They are (a) The limited programs that offer structured and routine on-the-job training for animal health workers; (b) Unequal distribution and inadequate quantity and quality of highly skilled workforce with appropriate technical training and scientific skills to combat public (and animal) health challenges at the frontline; (c) Health challenges occasioned by climate change and drought, including feed, and water scarcity; and (d) Inadequate contingency, preparedness, and response planning for effective deployment of ready-to-trigger workforce capacity. In-Service Applied Veterinary Epidemiology Training (ISAVET) is a four-month long training program targeted at capacity building of frontline animal health professionals. The training, which is currently implemented in 17 African countries, is innovative and a customized field epidemiology program, which responds to specific needs in animal health and contribute to approaches utilizing One Health.

**Results:**

Several trainees have marked mindset change as shown in the outputs and outcomes. Positive attitudes towards improving animal health surveillance were noted during the evaluation process.

**Discussion and Conclusion:**

Most existing workforce capacities in the animal and public health systems were built for specific fields, and hardly respond optimally for cross-sectoral purposes. We proposed customised in-service applied veterinary epidemiology training that bypasses narrow-scoped workforce development but meets multifunctional, multidisciplinary and multisectoral needs before and during emergencies.

## 1. Introduction

The International Health Regulation 2005 (IHR 2005) guidelines recognizes the need for World Health Organization (WHO) member states to meet their core capacity requirements for surveillance, reporting and response activities (1). Using the whole of society approach to mobilize multiple sectors and disciplines to work together in fostering well-being and tackling threats to health and ecosystems is important. This approach must address critical societal needs, act on climate change mitigation, and contribute to sustainable development and health systems at national and subnational levels (2).

The JEE is a voluntary, collaborative, multisectoral process to assess country capacities to prevent, detect and rapidly respond to public health risks. Kenya participated in the Joint External Evaluation (JEE) in February to March 2017 (3). In the follow-up report, the lack of a structured and routine on-job training for animal health workers was identified as a major hindrance to attaining workforce with appropriate technical training and scientific skills to combat public (and animal) health challenges at the frontline (3). Furthermore, Kenyan battles persistent challenges that need innovative solutions including: (a) Limited Programs that offer structured and routine on-the-job training for animal health workers (4, 5); (b) Unequal distribution and inadequate quantity and quality of highly skilled workforce with appropriate technical training and scientific skills to combat public (and animal) health challenges at the frontline (4); (c) Health challenges occasioned by climate change and drought, feed, and water scarcity (6, 7); and (d) Inadequate contingency, preparedness, and response planning for effective deployment of ready-to-trigger workforce capacity (8, 9).

The animal health services in Kenya are decentralized into the national and county (subnational) services, and these are led by veterinary doctors, and supported by animal health scientists, technicians and other veterinary paraprofessionals. The dearth of veterinarians (quantitatively) are observed in the government ministries, para-veterinarians constituted the majority animal health workforce in Kenya, both at the national, counties, and the private companies. The capacities of this workforce to respond, prevent and detect disease specific zoonoses and One Health concerns range from 1.17 to 3.25; 1.19 to 3.44, and 1.14–3.67 respectively, on a scale of 5.00 (10). This is linked to the uneven level and intensity of training among the different categories that form the animal health workforce, as well as available resources. Hence, these challenges warrant that innovative methods are used to address them, and with the aim of capacity building of frontline animal health workers with the requisite skills to enhance early detection and response in mitigating the impact of potentially transboundary and zoonotic pathogen threats at their source was determined as issue of high priority (11, 12).

To respond to these challenges, the Food and Agriculture Organization of the United Nations (FAO), the Institute for Infectious Animal Diseases (IIAD) of Texas A&M University, through funding from the United States Agency for International Development, developed a comprehensive curriculum (13), for Frontline In-Service Applied Veterinary Epidemiology Training (ISAVET). The curriculum that was launched in a pilot training in October 2018 to April (13) in Kampala Uganda has since been rolled out in 17 countries of West, Central, East and Southern Africa. This training program primarily focuses on building the capacity of field animal health workers particularly veterinarians and paraveterinarians who are critical in addressing endemic, emerging infectious and transboundary animal diseases on the frontline. It also deliver additional benefit by providing workforce that meets the cross-sectoral collaboration strengthening by focusing on One Health training in field epidemiology to enable common understanding between public and animal health workforce, because structurally, it complement the Field Epidemiology and Laboratory Training Program (FELTP). In addition, because it is necessary to have mindset change to deliver impactful animal health services at the frontline, the learning of new skills and thinking around health service delivery were included using the concept that focuses on paradigm shift in service delivery. In countries, FAO works with the relevant ministries responsible for agriculture, health, wildlife and environment to implement the training Program.

In Kenya, the first Cohort of ISAVET training was launched on 7th June 2021, in Nakuru County and to date, three cohorts have been trained. Specifically, the ISAVET in Kenya is targeted at building capacities in 8 domains, 14 core competencies and 47 skills developed using a consultative approach among stakeholders [Supplementary Table S1; (13)]. The training consisted of 4 weeks of classroom training (3 weeks of didactic and 1 week of field experience). This was followed by 12 weeks of implementation of field activities relevant to the areas where trainees originated from.

### 1.1. Description of the in-country ISAVET program

#### 1.1.1. Preparation for ISAVET training

##### 1.1.1.1. Building national consensus and establishment of national ISAVET coordination structures

To facilitate the adoption of the training program in-country, FAO together with the relevant national partners organized a pre-inception stakeholders’ consultative meeting to introduce the ISAVET program and get buy-in from the national and subnational governments. A comprehensive stakeholder mapping and analysis was also conducted in consultation with the State Department of Livestock’s Directorate of Veterinary Services (DVS) and the Zoonotic Disease Unit (ZDU). This process led to the identification of relevant stakeholders by institutional domain (major government stakeholders, minor government stakeholders, regulators, research institutions, partners/funders) and their area of interest (influence) (Supplementary Table S1).

Important stakeholders were mandated to nominate representatives to the *National ISAVET Program Steering Committee* (NIPSC), a body that is chaired by the Director of Veterinary Services. Other members of the NIPSC were from the Ministry of Agriculture, Livestock, Fisheries and Cooperatives (MoALFC), Ministry of Health, Kenya Wildlife Services, Council of Governors, Academia, Development Partners, Regulatory and Professional Bodies. The NIPSC operates through a terms of reference that include the provision of strategic guidance and support, and the monitoring and evaluation of progress and impact of the ISAVET program at country level.

Furthermore, a team of professionals were carefully selected to form the ISAVET Technical Working Group (ITWG), including staff from the MoALFC, ZDU, County Veterinary Services, Academia, Research and Development Partners. The terms of references for the ITWG was to provide advice and support in planning, preparation and implementation of the frontline ISAVET course and the provision of oversight of trainer and mentor competencies and skills. In preparation for the 1^st^ cohort of ISAVET training, the global curriculum was first contextualized to Kenya, endorsed and validated by national stakeholders in November 2020. In the context of ISAVET, The trainers were persons who are technically sound in matters of epidemio-surveillance, and who were involved in the one-month didactic training of ISAVET trainees. The mentors were either academic or institutional mentors. The academic mentor provided advice to trainees on technical matters in epidemiology for the three-month field activities while the institutional mentor assisted in the design of a realistic and achievable study and played oversight during the three-month field study period.

##### 1.1.1.2. Planning and organization of the in-country ISAVET training

To implement the holistic training and enable mentoring approach of the ISAVET program, three training components were conducted. These included the 4-month in-service training program for frontline veterinarians and paraveterinarians, and a separate two-week long training of trainers and training of mentors workshops. The *National ISAVET Coordinator*, a former a graduate of the Field Epidemiology and Laboratory Training (FELTP), and a staff from the Directorate of Veterinary Services, coordinated the ISAVET program on behalf of the government of Kenya. The National ISAVET Coordinator worked closely with the *FAO-ECTAD National Epidemiologist*, who coordinated and facilitated the ISAVET program from FAO Kenya (Supplementary Figure S1).

##### 1.1.1.3. Training of mentors and trainers

In preparation for the rolling out of frontline ISAVET Program in Kenya, 10 mentors and trainers were trained in addition to the 5 trainers and 5mentors initially trained in the regional training workshop held in Nairobi, Kenya in October 2018. Following an open call, a competitive process was utilized to select qualified individuals from the national and sub national veterinary services, the Meat Training Institute and the Kenya Wildlife Services. Briefly, the potential trainers and mentors were taken through a 5-day training on specific roles, responsibilities and required competencies expected to support the trainees during the 4 months applied veterinary epidemiology training. Content of the training included exercises on the technical requirements to ensure successful trainee program until completion, the curriculum and case study development tools, the monitoring and evaluation tools, core dimensions of the trainer-trainee and trainer- mentor relationship and trainer responsibilities and expectations. This specific training was facilitated by mentors and trainers who had an initial training in the regional pilot workshop for trainers and mentors held in Nairobi in October (13). The national training was based on the approved curriculum adopted by the NIPSC.

##### 1.1.1.4. Scoping mission

A key pre-course activity in planning for the frontline ISAVET training workshops was the scoping mission exercise. The goal of the mission was to assess the suitability of the selected County (subnational facility) to host ISAVET training of frontline health workers with the specific objectives of determining the prevalent disease(s) present at the County that can be used for field activities; identifying the specific location of field activities, and preparing and locating available resources suitable for the field activities. Statutorily, the scoping mission team was led by the government ISAVET focal person, but was inclusive of officers from FAO, and DVS, MoALFC. During the mission, consultations were made with the various stakeholders using a guided scoping mission tool that comprises of the stakeholder consultation checklist, field planning and laboratory planning tools. The scoping mission reports was shared with the stakeholders (FAO country team, the DVS and the respective counties that should host the month-long didactic training). The reports informed the comprehensive follow up administration and logistic actions, communication and other preparations.

##### 1.1.1.5. Trainees selection and preparation for the training

The calls for application of potential veterinary and paraveterinary trainees from national and sub national veterinary services and institutions were sent out twice *per annum* (2 months before the commencement of the training) to all animal health and public health networks. Applications to the ISAVET training were done through the standardized ISAVET application form, and each application must be endorsed by respective County Director of Veterinary Services (CDVS) and the immediate work station supervisor. The CDVS was the designated as the Institutional Mentor during the field-based training. Using a pre-defined set of criteria, all applications were vetted by the 10-member selection committee representative of the DVS (Veterinary Epidemiology and Economics Unit and Training division), the ZDU and Council of Governors (CoG). From a set of applications, only 25 successful applicants were selected to constitute a cohort of ISAVET trainees. Each applicant was then pre-notified of the success of his or her application and informed of the venue of training, reporting dates, content and duration of the training, required proposed field case study concept note, and the necessary items to bring along to the training.

Approximately 4 weeks before the training, proposed weekly lessons were assigned to trainers considering each trainer’s competency, experience, and availability during the didactic duration of the training. Trainees were also matched to mentors based on geographical location of the trainees and mentors. Virtual meetings were held between the course coordinators at DVS and FAO with the trainers to go through the developed lessons, exercises and contextualize case studies as required. The mentors and trainees also have virtual and phone call engagements to refine the proposed field case study concept notes.

#### 1.1.2. Implementation and delivering of frontline ISAVET training

The trainees were introduced first to the concept of importance of mindset change and its output in reference to field epidemiology following training. On weekly basis, trainees were taken through courses in epidemiological surveillance, field investigations, preparedness, disease prevention and response. Other topics covered included communication, ethics, and professionalism during disease investigation. Furthermore, trainees were taken through training on collaborative engagement and shared resources and modalities for implementing field project using the One Health perspectives. The didactic portion of the training were delivered over 4-week period structured as outlined in the Supplementary Table S2a and Supplementary material S2b.

## 2. Materials and methods

Using biodata provided by trainees and the geo-coordinates of the place of primary assignment of each trainee, the spatial–temporal maps of the ISAVET field implementation for the three cohorts were conducted. All serial maps were created using QGIS v3.3^1^.

Additional data used to evaluate the ISAVET program and trainees were collected using mixed method (quantitative and qualitative). Specifically, three surveys (plus four supplementary questionnaires) were designed and pretested among three technical staff (see Supplementary Appendices S1–S7). Based on the feedback, the questionnaire was modified to ensure consistency, clarity and validity, and coded on Google forms. The final version was self-administered online, or the links for the online surveys were shared *via* email and social media (WhatsApp). Responses on (1) Self-reported comparison of pre and post-training survey among ISAVET trainees; (2) Scores for all areas of competencies and veterinary skills assessed before and after the 4 weeks didactic training; and (3) Scores for areas of intended or ongoing applications of veterinary epidemiology skills, and perceptions on the most useful veterinary epidemiology skills post-training were captured on a Likert scale (i.e., 1 = strongly disagree, 2 = disagree, 3 = neutral/no change, 4 = agree and 5 = strongly agree). Other responses were either dichotomous in nature or non-structured (qualitative). Whereas the pre-training and post-training practices surveys were carried out among all the 76 ISAVET trainees, the 6-month post training practices survey was only carried out to target only the 25 ISAVET Cohort 1 trainees.

## 3. Results

**Figure 1:**
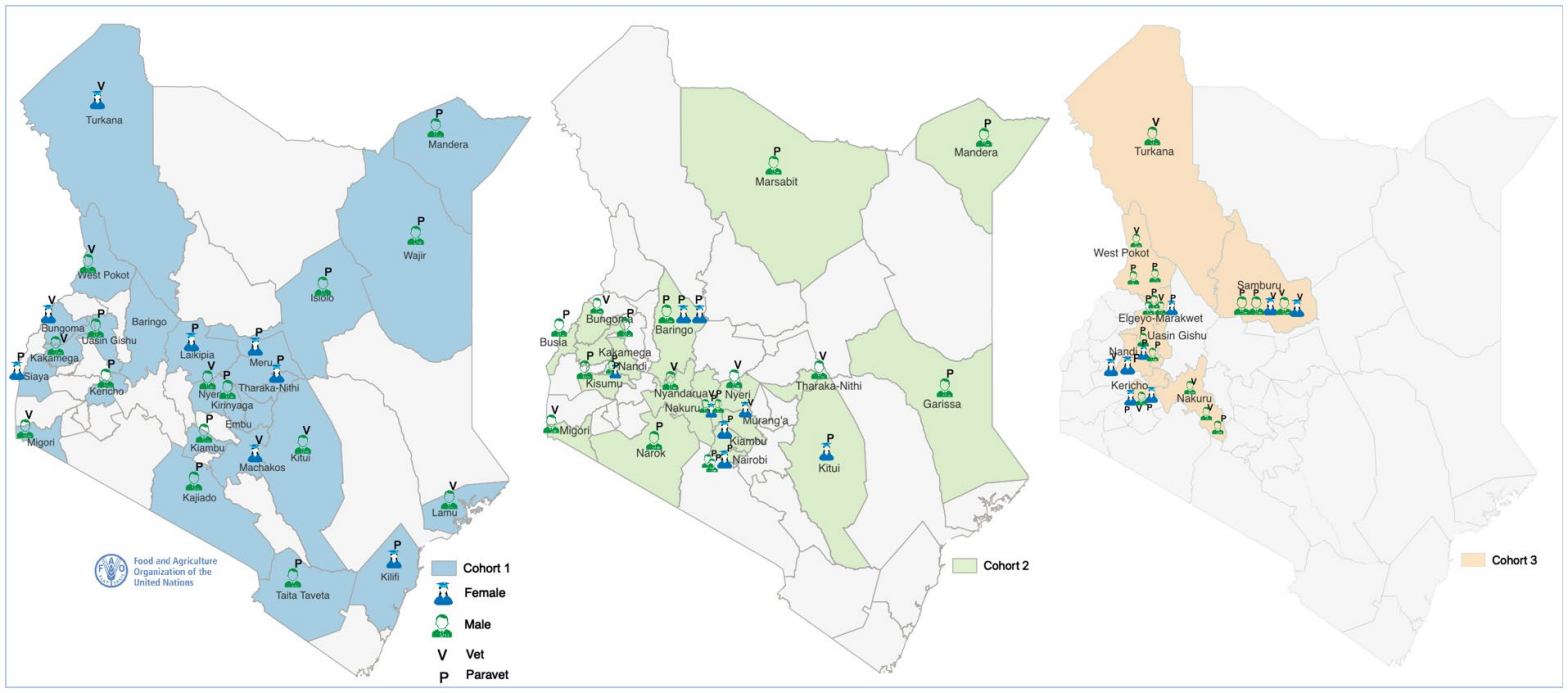
Spatial spread of trained cohorts of in-service applied veterinary epidemiology training (ISAVET). Note that Cohort 1 is represented in (blue), Cohort 2 (green), and Cohort 3 (light brown).

In total, 75 trainees have undergone the in country ISAVET program in Kenya to date with approximately 67% male and 33% female (Table 1), in addition to the three originally trained through the pilot Program in Uganda, 2018. Over 93% of the trained workforce are direct frontline officers in the subnational system. A total of 40/47 (85.1%) of the counties have been represented in the training, although the number trained per counties varied (Table 1 and Figure 1). The high animal production (HAP), the Arid and Semi-Arid lands (ASALs) and the North Rift Economic Bloc (NOREB) regions of Kenya have been covered to date. Participants have been drawn from county veterinary services (including from 6 border counties), the Meat Training Institute, a Regional Veterinary Investigation Laboratory, a border post (point of entry), a national food processing and export facility, and the Kenya Wildlife Services (KWS) (Table 1).

**Table 1:** Clustering of participants including the areas of routine practices and activities.

Criteria	Cohort 1	Cohort 2	Cohort 3*
Focused region of Kenya	Random nationwide	Random nationwide	North Rift Economic Bloc (NOREB)
Number of counties involved	24 (inclusive of 6 border counties and a Meat Training Institute)	22 (inclusive of one Regional Veterinary Investigation Laboratory, a point of entry and one national food processing and export facility)	9 (inclusive of the Kenya Wildlife Services (KWS) and Regional Veterinary Investigation Laboratory).
Description of areas covered	The high animal production and Arid and Semi-Arid regions of Kenya.	The high animal production and Arid and Semi-Arid regions of Kenya.	Arid and Semi-Arid regions in the North Rift Economic Bloc (NOREB)
Numbers in the Cohort	25	25	25
Period of implementation (Classroom didactic)	7 June, 2021 to 2 July 2021	7 March 2022 to 1 April 2022	6 June 2022 to 1 July 2022
Period of implementation (3-month Field work)	5 July, 2021 to 24 September 2021	4 April 2022 to 24 June 2022	4 July 2022 to 23 September 2022
Male: Female ratio	16 males: 9 females	17 males, 8 females	17 males: 8 females
Professional diversity of trainees	10 veterinarians, 15 paraveterinarians	8 veterinarians, 17 paraveterinarians	11 Veterinarians, 14 Paraveterinarians
Areas of work	24 County disease surveillance officers, (8 serving in border counties), 1 officer from the Meat Training Institute who is now posted at border inspection point.	22 County disease surveillance officers, (4 serving in border counties), 1 border control officer, 1 veterinarian at the Regional Veterinary Investigation Laboratory and (1 paraveterinarian at the national food processing and export facility)	24 County disease surveillance officers, (3 serving in border counties), I veterinarian at Kenya wildlife service s and 1 veterinarian at the Regional Veterinary Investigation Laboratory

Cohort 3: classwork is completed and field component is ongoing. The list of specific topics covered to date are available in the Supplementary Table S2.

**Figure 2:**
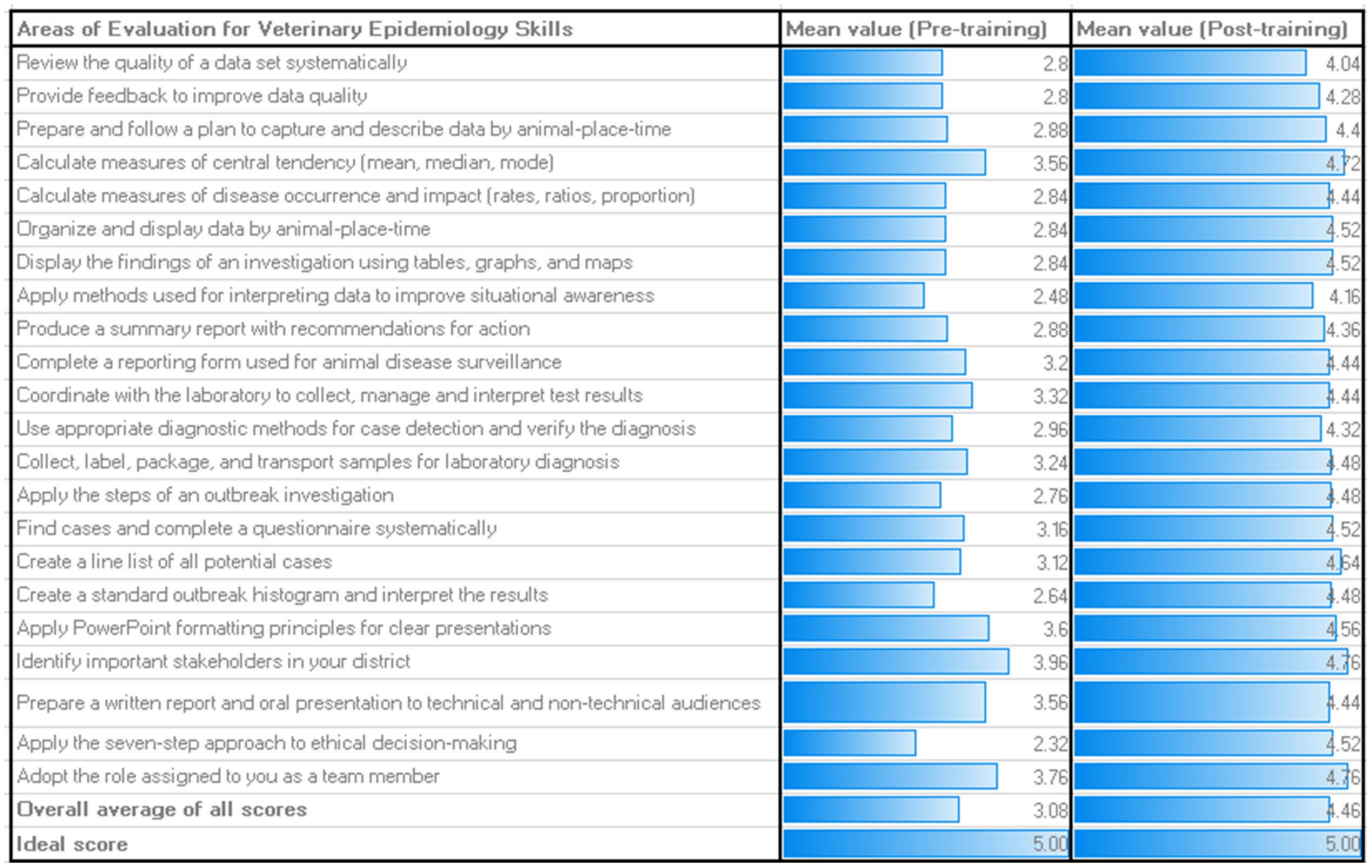
Mean scores for all areas of competencies and veterinary skills assessed before and after the 4 weeks didactic training. Only 65% of the trainees understood the key One Health concepts at the beginning of the didactic training but this increased to approximately 88% during the post-training practices survey.

The trainees included 33 veterinarians (44%) and 42 paraveterinarians (56%). On the specific areas of operations, 70 of the trainees operate as County disease surveillance officers (93.3%), including 14, who work in the border counties, and one representative each as meat training officer, border control officer, laboratory veterinarian, national food processor at the export facility and a wildlife veterinarian (Table 1). The trainees have had marked mindset changes as shown in their positive attitudes towards improving animal health surveillance in their localities and based on the outcomes of the evaluation process. For instance, positive attitudinal shift and commitments were seen in the mean gain of the veterinary epidemiologic skills of the ISAVET graduates [increase of at least 44.9%: ((4.46/3.08 *100) – (3.08/3.08 *100))] (Figure 2), and the confidence in field implementation, field applications of epidemiologic skills and One Health approach all improved (Table 2).

**Table 2:** Self-reported comparison of pre and post-training survey among ISAVET trainees.

Criterion to measure attitude, confidence and commitment to epidemio-surveillance capacities	Pre-training average (*n* = 25)	Post-training average (*n* = 25)	Value of *p*	*Χ^2^*
Map value chains with stakeholders during peace time	3.48	4.56	0.06	3.6
Contribute to post-incident assessments by validating and providing field level data	2.76	4.4	0.01	6.5
Based on the ISAVET curriculum I have been introduced to, ISAVET will help me gain skills in field epidemiology	4.76	4.92	0.52	0.4
Participating in ISAVET will be important for my professional development	4.92	5.00	0.53	0.4
I will use what I will learn from ISAVET in my job	4.96	4.88	0.66	0.2
I am only going to participate in ISAVET because my supervisor asked me to	1.44	1.12	0.61	0.3
I know what is expected of me after I complete the course	4.44	4.84	0.28	1.2
I believe that ISAVET will help me develop professionally	4.92	4.96	0.80	0.1
I am eager to apply the knowledge I will gain in ISAVET in my job	4.96	5	0.66	0.2
ISAVET will improve my field epidemiology skills	4.96	4.96	1.00	0.0
My expectation from the ISAVET training is to able to improve quality of data and reports	4.92	4.96	0.80	0.1
I am confident my supervisor/management will provide me with the necessary support to apply the skills I learnt through ISAVET in my daily work.	4.64	4.64	1.00	0.0

The maximum score obtainable in the pre and post-training evaluation is 5.0.

On a scale of 1–5, the mean value of the veterinary epidemiologic skills of the trainees at the beginning of the training was 3.08/5.00 (61.6%), which increased to 4.46/5.00 (89.2%) by the end of the training (Figure 2). At the pre-training level, the three skills with the highest scores were: (a) identifying important stakeholders in the locality (district) (3.96/5.00; 79.2%); (b) adopting roles assigned during field activities as a team member (3.76/5.00; 75.2%); and (c) calculating the measures of central tendency (mean, median and mode) and preparing a written report and oral presentation to technical and non-technical audiences (3.56/5.00; 71.2% each) (Figure 2). The weakest skills at the pre-training stage were the application of the seven-step approach to ethical decision-making (2.32/5.00; 46.4%) and the application of methods used for interpreting data to improve situational awareness (2.48/5.00; 49.6%) (Figure 2). To highlight a few, at post-training level, the three strongest scores remained identifying important stakeholders in the locality (district) and adopting roles assigned during field activities as a team member (4.76/5.00; 95.2%), and calculating the measures of central tendency (mean, median and mode) (4.72/5.00; 94.4%) (Figure 2).

**Figure 3:**
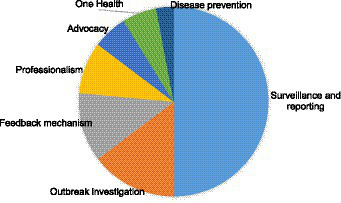
Plans on how ISAVET knowledge and skills will be utilized post-training.

On the likely immediate post-training application of epidemiologic knowledge and skills gained during the didactic training, 50% indicated willingness to apply their knowledge to improve surveillance and reporting, while 14.7 and 11.8% wanted to improve outbreak investigation and feedback mechanisms, respectively. Only 2.9% indicated the utilization of the new knowledge to improve disease prevention (Figure 3). Approximately 8.8% believed that the new knowledge could improve their professionalism and 5.9% each believed that it should improve advocacy and applied One Health (Figure 3).

**Figure 4:**
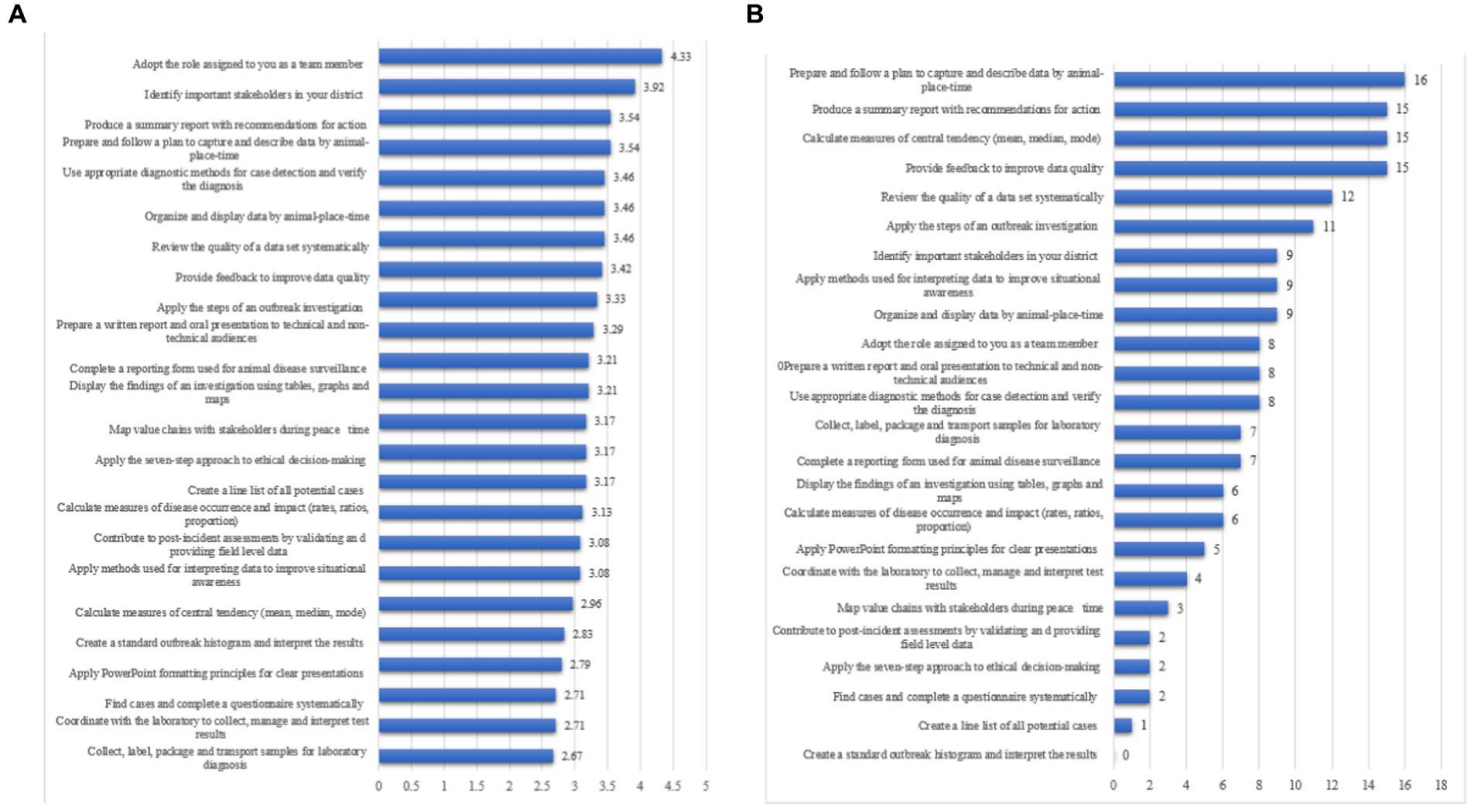
**(A)** Mean scores for areas of intended or ongoing applications of veterinary epidemiology skills, and **(B)** perception of trainees on the most useful veterinary epidemiology skills gained, 6 months post-training.

Six-month post training, 86.6% of the trainees were adopting roles assigned to them during field activities as team members, 78.4% were identifying important stakeholders in the locality (district) and 70.8% each were still producing summary reports with recommendations for action, and preparing and following up plans to capture and describe data by animal, place and time (Figure 4A). Six-month post training, trainees perceived that the most useful veterinary epidemiology skills gained were: preparing and following up plans to capture and describe data by animal-place [16/25 (64%)], and producing summary reports with recommendations for action [15/25 (60%)]. None of the trainees perceived that creating a standard outbreak histogram and interpreting the results were as useful as other skills (Figure 4B).

Furthermore, in terms of attitude, confidence, and commitments, 12 areas were evaluated, and the trainees gained marginal increases in some areas (*p* > 0.05) and only in the area of contribution to post-incident assessments by validating and providing field level data were significant gain observed (*p* = 0.01) (Table 2).

## 4. Discussion

In this work, the implementation of ISAVET was explored in Kenya and showed how international development organization and national partners can collaborate and co-create to build the national workforce with resultant positive effects on country’s epidemio-surveillance systems, with reflections on the One Health approach. The need for such trained workforce cannot be underestimated (13). Specifically, in the delivery of ISAVET Program in Kenya, only 78 trainees have completed the training in three cohorts, but the Program intends to generate additional 50 participants *per annum*, unless the scaling up is facilitated by other partners. It is acknowledged that the current numbers being trained *per annum* is inadequate in a country like Kenya, where skilled workforce demand is huge, and animal resources are enormous. While it is understood that this work is almost completely externally funded, it is acknowledged that there is a need to ramp up local and national resources to rapidly expand the pool of trainees and place them where they are most needed in order to meet the epidemio-surveillance needs of Kenya. Over-dependency on externally sourced funds have been identified as a limitation to scale up the implementation of best initiative in countries of Africa (14–17).

The spatial spread of trainees from counties and where they are working appeared balanced; this covers 85% of the geographical landscape; however, the number of trained personnel per county range from 1 to 5, figures that need to be up-scaled. Perhaps a training of trainer approach can be utilized to fast-track the trained personnel per county, through the extended training of officers at the county level by the ISAVET graduates. Future training can thereafter be prioritized through upskilling of these locally trained individuals. From the third cohorts, the focus of selecting trainees has been re-organized from having a national spread to prioritizing a region in each cohort. The change was occasioned by the different challenges and needs that exist in the different zones. For instance, the ASALs faces challenges of drought, with resultant loss of livestock resources due to lack of water and feed scarcity. Such special needs are therefore considered in packaging regional-level training and discussed alongside the training of the cohorts. Localizing animal and public health solutions have been considered beneficial than a generalized approach (18). This observation is important because it brings the veterinary services in direct contact with the people who need the services, and reduce the critical response time to public health and animal health events. The benefits of reducing critical response time have been emphasized previously (19). This also have implications on One Health at local level as animal health issues often result human health in the frontlines, e.g., anthrax, rabies, brucellosis and trypanosomiasis among others.

Important observations were the attitudinal shift especially in the mean veterinary epidemiologic skills, which increased by 44.9% and the observed boost in confidence in field implementation, field applications of epidemiologic skills and One Health approach. These is important for professionalism and effectiveness of the frontline officers. The training highlighted the weakest areas of skills of the frontline officer and provided feedback to the training institutions (universities and higher colleges). The follow-up monitoring and evaluation was particularly useful because it tracked each training in real time and served as additional evaluative tool in service delivery and career progress of animal health workforce. Although the training exposed the trainees to a two-way (epidemiology-laboratory) and four-way (epidemiology-laboratory in public health and animal health) linkages, and the direct supervisors of the graduates understood the skills and knowledge gained by trainees, the CDVSs were somewhat disconnected from the program thus necessitating a need to introduce executive session of the program.

It is confirmed that the current ISAVET graduates have reached beyond the limited scope of epidemio-surveillance in animal health alone, but some have contributed to or have been utilized for support services where public health officials are in short supply, for instance, during the surge in COVID-19 epidemics, and during county-level unusual public health events or incidences needing One health approach. Data-driven approach in animal health and broad training in animal health for the benefit of public health have been emphasized (20, 21). It should be understood that most African states have inadequate capacity (number and quality) of animal and public health workforce to meet the immediate animal and public health challenges. In the recent COVID-19 challenges, countries like Ghana, Liberia, Senegal, Sierra Leone, Tanzania, and Nigeria utilized the veterinary laboratories and its workforce to increase diagnostic capacities and contribute to response to COVID-19 outbreak when the public health laboratories were over whelmed (22–24).

Basically, the ISAVET program has addressed some of the originally identified challenges mentioned in the introduction above: (1) It is offering a structured and routine on-the-job training for animal health workers; (2) Although, it cannot even out the unequal distribution and inadequate in quantity of highly skilled workforce, it has shown capacity to improve the quality and effectiveness of this workforce through appropriate technical training and scientific skills to combat both animal health and public health challenges at the frontline; (3) The training also capacitated innovative thinking to resolve associated health challenges of climate change and drought; and (4) It has also contributed significantly to improving contingency, preparedness, and response planning for effective deployment of frontline rapid response team members.

Finally, the field interventions and follow-up reports have presented both the national and subnational governments with opportunities to prioritize locally significant disease situations, level of awareness, knowledge and practices, and skill levels with regards to zoonotic, emerging, re-emerging and transboundary diseases, as well as other issues including antimicrobial resistance and One Health.

In conclusion, valuable capacities already exist in the animal and public health systems in Africa. These capacities were built for specific fields in isolation, and hardly are made available for other purposes. This lack of integration is costly, though these costs are under-appreciated most times. By implementing some minor adjustments and additional surge training that programs like FELTP and ISAVET are doing, such originally trained workforce can be upskilled to meet multifunctional needs including addressing emergencies, when they arise. Because, emergency does not allow much room for preparation when it occurs, these surge capacities must be prepared ahead, and can be rapidly adjusted to meet need when necessary.

## Data availability statement

The original contributions presented in the study are included in the article/Supplementary materials, further inquiries can be directed to the corresponding author.

## Author contributions

RS, KC, SO, CL, ON, and FF coordinated the research implementation. RS, MN, SO, HO, MA, JO, and TO conducted the training. CL, ON, CB, and FF played oversight roles. RS and FF did the original manuscript draft. ET, RS, and KC conducted the monitoring and evaluation. All authors contributed to the review and finalization of the draft.

## Conflict of interest

The authors declare that the research was conducted in the absence of any commercial or financial relationships that could be construed as a potential conflict of interest.

## Publisher’s note

All claims expressed in this article are solely those of the authors and do not necessarily represent those of their affiliated organizations, or those of the publisher, the editors and the reviewers. Any product that may be evaluated in this article, or claim that may be made by its manufacturer, is not guaranteed or endorsed by the publisher.

## Footnotes

^1^ https://www.qgis.org/en/site/
