# Supplementary material for: Building subnational capacities in animal health to deliver frontline cross-sectoral health services in Kenya
Source: Front Vet Sci. 2023 Aug 4;10:1150557. doi: 10.3389/fvets.2023.1150557 (PMC10436308; doi:10.3389/fvets.2023.1150557)
Supplement: Supplementary file 1 [file Data_Sheet_1.docx]

Supplementary Material

Article Title

**Rinah Sitawa, Evans Tenge, Khadija Chepkorir, Mark Nanyingi, Sam Okuthe, Caryl Lockhart, Harry Oyas, Obadiah Njagi, Mary Teresa Agutu, Jack Omolo, Tequiero Okumu, Charles Bebay, Folorunso O. Fasina**

*** Correspondence:** Corresponding Author: folorunso.fasina@fao.org

# Supplementary Data

**Supplementary Table S1**. Composition of National ISAVET Program Steering Committee and the Technical Working Group

| **National ISAVET Program Steering Committee** | | |
| --- | --- | --- |
| **Institution/ organization** | **Section** | **Number of representatives** |
| Ministry of Agriculture , Livestock, Fisheries and Cooperatives (MoALFC) | Principal Secretary - State Department of Livestock | 1 |
|  | Directorate of Veterinary Services (DVS) | 2 |
| Ministry of Health | Department of Preventive and Promotive Health. | 1 |
| Kenya Wildlife Services | Veterinary Capture, Forensic and Molecular Lab Services | 1 |
| Council of Governors | Agriculture Caucus | 1 |
| Academia | University of Nairobi | 1 |
| Development Partners | Food and Agriculture Organization | 2 |
|  | Non-governmental Organizations | 1 |
| Regulatory and Professional Bodies | Kenya Veterinary Board (KVB) | 1 |
|  | Kenya Veterinary Association (KVA) | 1 |
|  | Kenya Veterinary Paraprofessional Association (KVPA) | 1 |
| Total | | 13 |
|  | | |
| **National ISAVET Technical Working Group** | | |
| Ministry of Agriculture, Livestock, Fisheries and Cooperatives | Zoonotic Disease Unit | 1 |
|  | Field Epidemiology and Laboratory Training Program | 1 |
|  | Training Division, MoALFC | 1 |
| County Veterinary Services | County Directorate of Veterinary Services (Mentor) | 1 |
| Academia | University of Nairobi | 1 |
| Research | Core Unit Trainer | 1 |
| Development Partners | Food and Agriculture Organization | 2 |
| Total | | 8 |

*Cohort 3: classwork is completed and field component is ongoing.*

**Supplementary Table S2a**. Generic Schedule of training for the four week didactic during the ISAVET training program

**
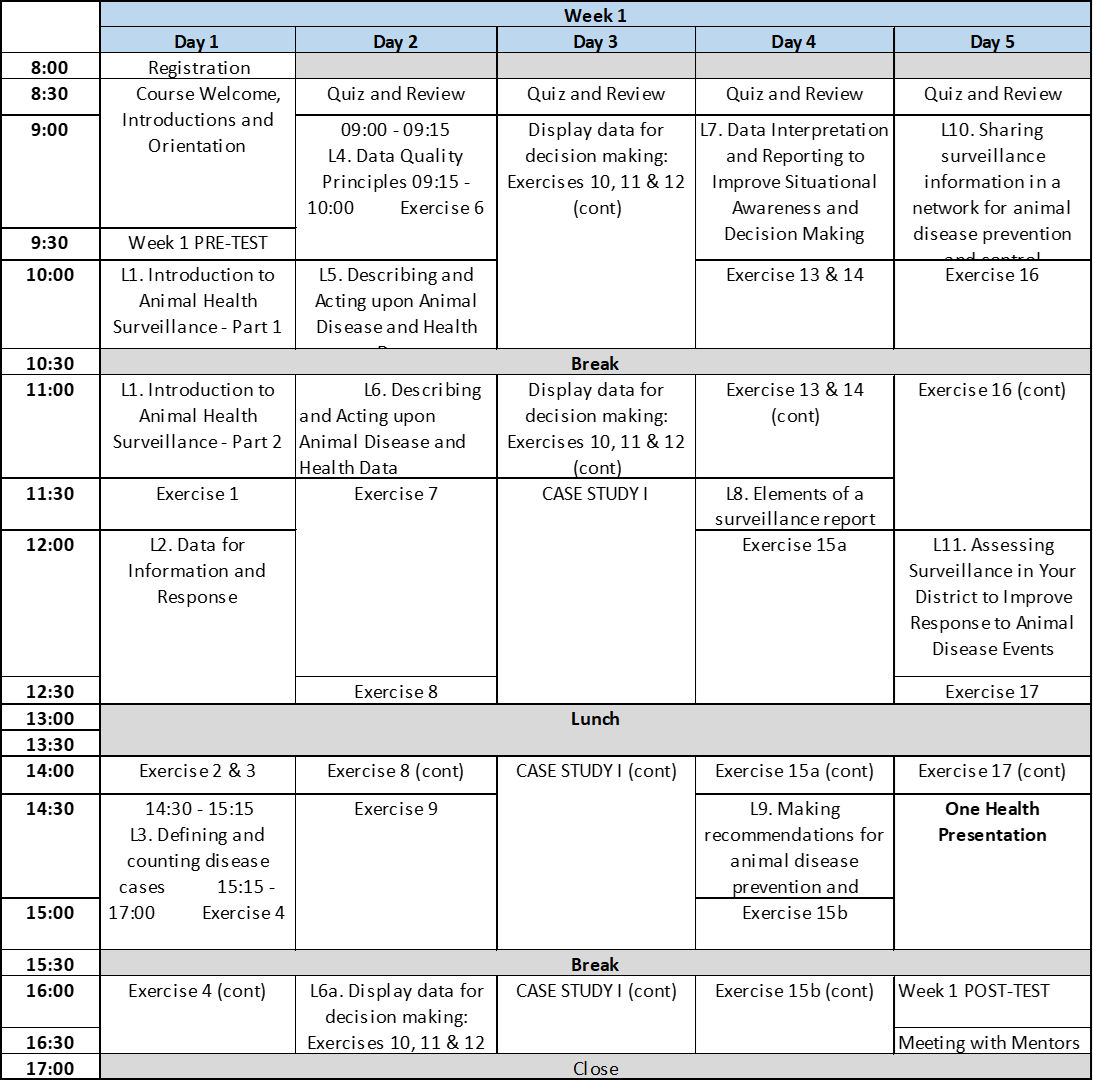
**

**
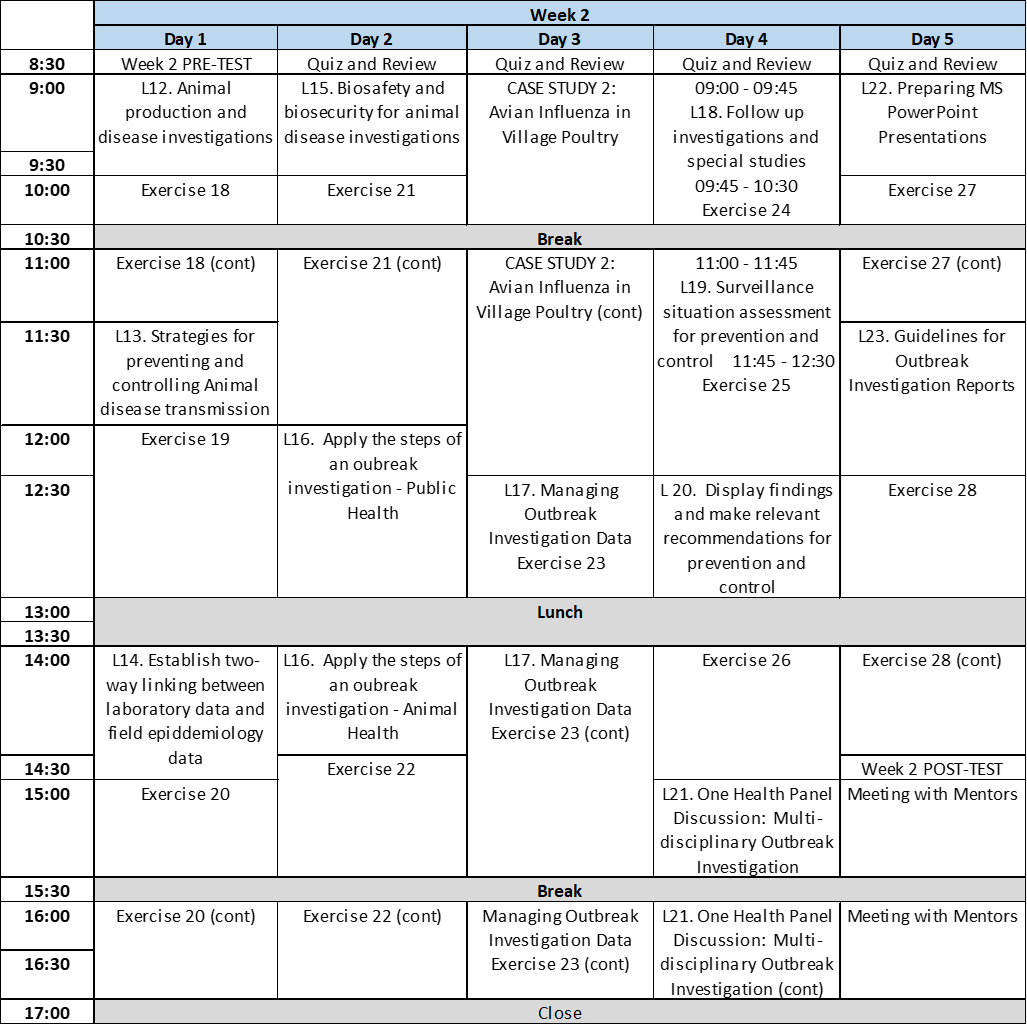
**

**
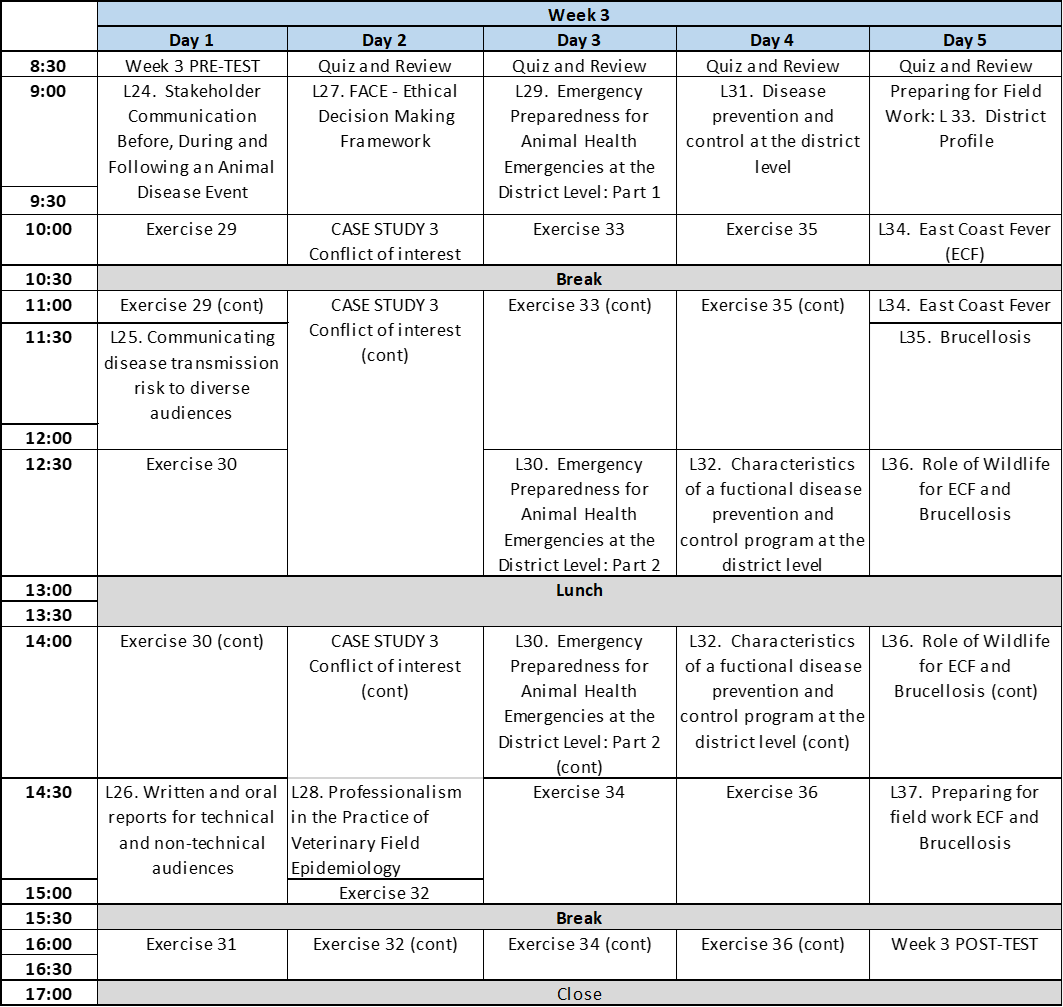
**

**
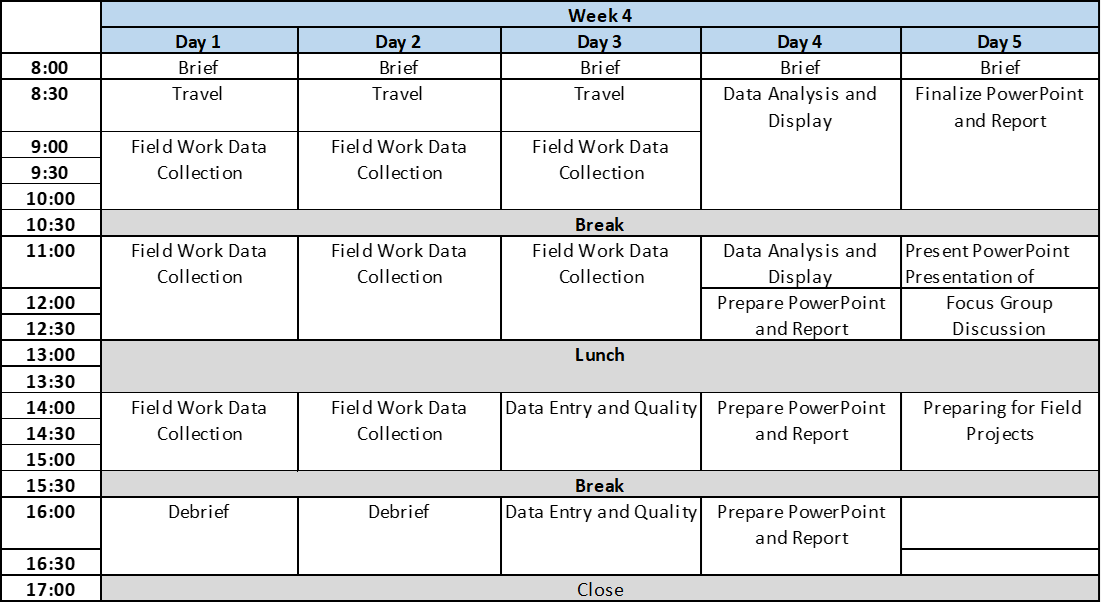
**

**Supplementary material S2b**. Detailed notes to accompany Section 1.1.2 (Implementation and delivering of Frontline ISAVET Training) for the four week didactic during the ISAVET training program

***1.1.2.1 Week 1: Focus on Epidemiological Surveillance***

Lessons, exercises, and case studies are presented to enable trainees define and differentiate surveys, surveillance, and field monitoring exercises. Components of an animal health surveillance system and flow and sharing of field data among stakeholders are described. Trainees are taken through skills to define and count disease cases in population and descriptive analysis of disease events by animal, place, and time. Exercises are conducted on the application of Microsoft Excel to calculate measures of central tendency e.g. mean, mode, measures of occurrence and impact such as attack rates and data description in terms of animal, place, and time. Data quality audit is also introduced during this week and integration of One Health is evaluated in each proposal and assignment (Supplementary Table S2).

***1.1.2.2 Week 2: Focus on Field Investigation and Response***

Training is primarily focused on epidemiological field investigations; transitioning from field to outbreak investigations and utilization of these investigations in managing animal production or disease events, value chain events, zoonoses, food safety and public health events, import and export events and wildlife disease events. Approaches for preventing and controlling animal disease transmission are introduced with discussions on strategies such as zoning and compartmentalization. A plenary session on One Health is also organized that brings together OH stakeholders from public health, animal health and the environment, including wildlife, and where necessary, ecosystem specialists. The trainees use part of the week’s program to work on their field proposals under guidance of the ISAVET trainers (Supplementary Tables S2 and S3).

***1.1.2.3 Week 3: Focus on Preparedness, Disease Prevention and Response, Communication, Ethics, and Professionalism***

These set of training exercises are meant to impart skills on identification and description of hazards, analysing risk and communicating to different audiences. The trainees carry out exercises where they identify the risk pathways for various diseases. Lectures and exercises on identification of important stakeholders, stakeholder mapping, risk communication, best practices in risk communication and message mapping for different audiences before, during and after disease outbreaks and message mapping are conducted. Trainees are also taken through professionalism and ethics in the practice of veterinary field epidemiology to understand the role of ethics in veterinary practice and how to recognize common ethical issues in veterinary practice. The trainees use the same opportunity to continue to work on their field proposals under guidance of the ISAVET trainers (Supplementary Tables S2 and S3). In order to refine their field case study proposals, the trainees make a 10–20-minute presentation to the trainees and trainers for a peer-to-peer trainee and trainer input into the proposed study. This provides more in-depth information on communication best practices for both oral and written reports. In addition, a review of the three weeks of training is conducted to prepare the trainees for the week four.

***1.1.2.4 Week 4: Field Work Training***

Trainees are taken through lessons and exercises to adequately prepare them for field work, an opportunity to practice skills taught during the preceding three weeks of class training. A more detailed review and simulation on biosafety and biosecurity training is covered emphasizing on appropriate dressing-up (donning) and dressing-down (doffing) procedures using personal protective equipment, where necessary in the farm areas. Before the week 4 field activities/intervention, trainees are teamed into 5 groups and are required to develop week 4 field concept notes and study proposals on priority diseases identified during the ISAVET Scoping mission in the host counties. These concepts are reviewed and approved by week 3 and 4 trainers before the week’s field intervention. During the field work, data are collected, analysed and summary reports prepared and presented by each of the 5 groups to stakeholders including farmers and policy makers on the last day of the didactic training. In addition, during the week, ISAVET trainees meet physically with the assigned ISAVET Mentors as the necessary first step to familiarize with each other, conclude on the field case study title and review on the standardized templates for weekly surveillance reports, data quality audits and field case reports. The didactic phase of ISAVET training thereafter closes with the immediate dissemination of field week training reports to the stakeholders and an overview of the required submissions during the three-month mentored trainings (Supplementary Table S2).

***1.1. 3 Monitoring, Evaluation and Learning***

To ensure compliance, a process of monitoring, evaluation, and learning (ME&L) is integrated into the program. This include the global and regional comparative evaluation as well as benchmarking process and a national ME&L process as described.

***1.1.3.1 Global and regional overview, comparative evaluation, and benchmarking***

*National monitoring, evaluation, and learning*: To implement national ME&L, two sets of implementation strategy is utilized including, a). Tracking of all trainees throughout the period of the four-month training using a Microsoft Excel^®^-based monitoring and evaluation dashboard to ensure compliance (Supplementary Figure S2). This tool is updated weekly until the completion of each cohort of trainees; b). Three surveys and a follow-up monitoring activity are implemented primarily to evaluate the impact of ISAVET program. This include the:

1. The pre-training practices survey that is carried out at the beginning of the four weeks of didactic training.
2. The post-training practices survey that is carried out at the conclusion of the four weeks of didactic training.
3. The 6-month post training practices survey that is conducted among the ISAVET trainees, six months after graduating from the ISAVET program, and.
4. The follow-up monitoring visits that continues to track progress of the ISAVET trainees and their career track.


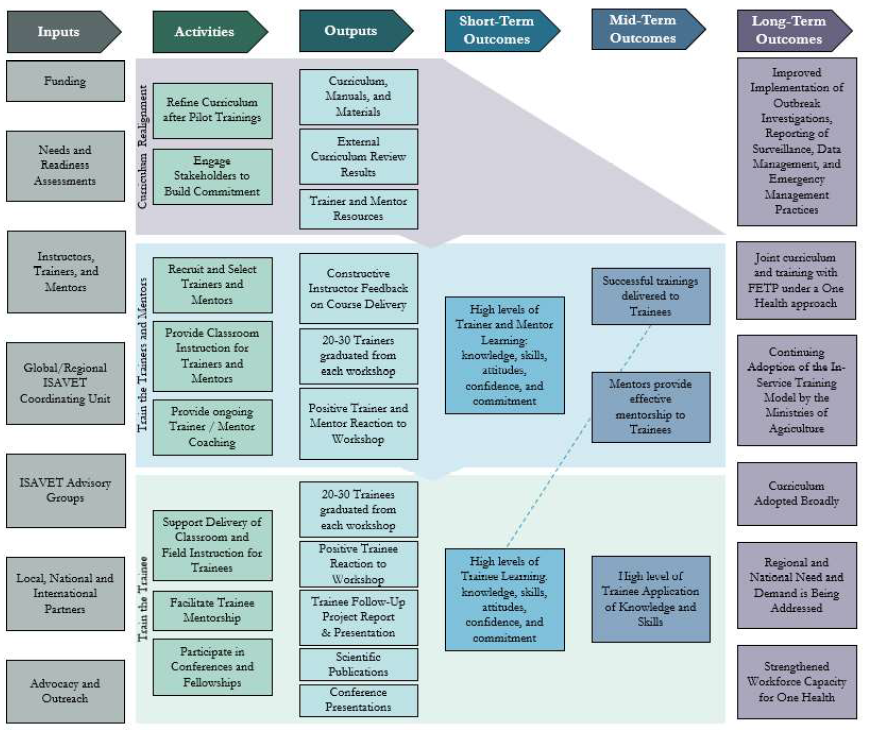


Supplementary Figure S1. Frontline ISAVET logic model used in the delivery of the training

**Supplementary Table S3**. Topics presented by the ISAVET trainees over the last three cohorts, 2021 - 2022

| ***Topics covered*** | ***Cohort 1*** | ***Cohort 2*** | ***Cohort 3**** |
| --- | --- | --- | --- |
|  | The Prevalence and Associated Risk factors of Peste des Petits ruminants (PPR) in goats in Lamu West Sub-County, 2021 | Prevalence of Fasciolosis and Associated Monetary Loss in Cattle, Sheep and Goats in Matayos Sub County Slaughterhouse, 2019-2021 | Retrospective data review on causes and occurrence of Bovine Mastitis in Milk samples submitted to Kericho Veterinary Investigation Laboratory 2019-2021 |
|  | Effectiveness of Peste des Petits Ruminants (PPR) Control Strategy in Sheep and Goats, West Pokot County, 2016-2020 | Surveillance on Organ Condemnations and Associated Financial Losses in Goats Slaughtered in Mwingi, Kitui County, 2022 | Retrospective review on Bovine Tickborne Diseases in Uasin Gishu County |
|  | Characterization of Occurrence of Lumpy Skin Disease in cattle, Kakamega County, 2018-2020, Kenya | Causes of Carcass and Organ Condemnation and Financial Losses in Sossion slaughterhouse Nakuru County, 2019-2021 | A retrospective review on causes of livestock organ and carcass condemnation and associated monetary loss in Turkana Central slaughterhouses 2019-2021 |
|  | Assessing Knowledge, Attitude and Practices of livestock keepers on Lumpy Skin Disease in Ugunja Sub County, Siaya County 2021 | Causes of Organ Condemnation and the associated Financial Losses in Cattle Slaughtered at Bungoma County Slaughterhouse, 2020-2021 | Assessment of Knowledge Attitude and Practices on Brucellosis among livestock keepers in Maralal ward, Samburu Central Subcounty July – September 2022 |
|  | Assessment of Knowledge, Attitude and Practices of Dairy Farmers to Foot and Mouth Disease and it’s Management in Maara Sub-County, 2021 | Prevalence and Financial Significance of Stilesia Hepatica in Sheep and Goats Slaughtered at Esageri Slaughter House in Eldama Ravine Sub-County, Baringo County from April to June 2022 | Knowledge, Attitude and Practices on the use of Veterinary Drugs and its association on Antibiotic Resistance in food producing animals in Samburu East Subcounty |
|  | Retrospective Study of Foot and Mouth Disease outbreaks in cattle in Laikipia County, 2013 – 2021 | Retrospective Study on Financial Losses due to Organs Condemnation Caused by Helminths infestation in Slaughterhouses in Transmara West, Narok County, 2022 | Retrospective review of dog bite cases in Humans in Nyiro ward January 2017-December 2021 |
|  | Assessment of Knowledge, Attitude and Practices of RVF among pastoralist communities in Lakoley-Basir ward, Eldas Sub- County 2021 | Prevalence of Enzootic Pneumonia in Pigs at Farmers Choice Slaughter House from selected farms in the month of April and May 2022 | Knowledge, Attitude and Practices about Rabies Prevention and control in domestic animals in Kipkelion West , Kericho County |
|  | Assessment of Knowledge, Attitudes and Practices on Rift Valley Fever among Pastoralist Communities of Mandera North Sub-County, Mandera County, Kenya between July, and September 2021 | The Occurrence and Community’s Knowledge and Perceptions on Hydatidosis in Slaughtered Ruminants, Emgwen Sub County, Nandi County April-June 2022 | A study on Knowledge, Attitude, Practices associated with Brucellosis among Pastoralists in Kiwawa ward, Pokot North June-September 2022 |
|  | Assessment of Rabies cases in domestic animals, Kericho Regional Veterinary Investigation Laboratory, 2016 - 2020 | Characterization of Rift Valley Fever Outbreaks in Livestock in Muranga County, 2018–2022 | Occurrence of East Cost Fever in Bovine in Uasin Gishu County, January 2020-2021 |
|  | Assessment of Animal Bite Cases and uptake of Rabies Post- Exposure Prophylaxis, Kilifi County, Kenya July 2020 - June 2021 | Assessment of Knowledge, Attitude and Practices and Canine Rabies Prevention and Control, Kasarani Sub-County, Nairobi April-June 2022 | Retrospective study on Prevalence, Occurrence and Economic Costs due to Fasciola and Stilesia infection of the livers of cattle, Sheep and Goats in Keiyo North Subcounty, July 2021 -June 2022 |
|  | A retrospective review of occurrence of clinical and laboratory Leishmaniasis in humans in Baringo County, Kenya, Jan 2016 to Jan 2021 | Retrospective analysis of Human-dog bite cases handled in Health Facilities in Mukurweini Sub-County, Nyeri County 2018 to 2021 | Knowledge, Attitude and Practices of Brucellosis among livestock farmers in Marakwet West County Sub County, July-September 2022 |
|  | Assessment of Knowledge, Attitude & Practices on Cattle Tick Borne Diseases in Wundanyi Mbale Ward, July – Sep 2021 | Characterization of human dog bites, temporal-spatial distribution and management, in Igambang’ombe sub County Tharaka Nithi County, 2018-2021 | Retrospective Review of Foot and Mouth Records in Regional Veterinary Investigation Laboratory , Nakuru 2016-2021 |
|  | Retrospective study on Occurrence of Liver fluke infestation in Uasin Gishu County, 2016-2020 | Investigation of Factors associated with Livestock Movement and Mapping of Common Routes in Eldama Ravine Sub-County, Baringo County | Knowledge, Attitude and Practices on Foot and Mouth Diseases among smallholder dairy farmers in Kiplombe ward, Uasin Gishu County, 2022 |
|  | Causes and Occurrence of Mastitis and in Cattle Milk Samples Submitted to Karatina Regional Veterinary Investigation Laboratory (RVIL), 2019-2020 | Assessment of the Priority Cattle Diseases, their Seasonal Occurrence and Impact on Livelihood in Mandera South Sub County, Mandera County (2022) | Major causes of Livestock Organs and Carcasses Condemnations and Associated Economic Losses in Keiyo South Subcounty 2019-2021 |
|  | Adherence to Antibiotic Withdrawal Period following Treatment of Dairy Cattle in Kirimari Ward, Embu County,2021 | Assessing Indigenous Knowledge, Attitude and Practices on Management and Control of Surra in Camel in Marsabit County, March- June 2022 | Review of Zoonotic Pathogens isolated in Kericho Regional Veterinary Investigation Laboratory 2016-2021 |
|  | Prevalence of Hydatidosis and Associated Monetary Loss in Slaughtered Cattle, Awendo Sub County | Assessment of Tick Control Practices among Cattle Farmers in Butere Sub-County, Kakamega, Kenya, 2022 | Characterization of Brucellosis Cases and Assessment of Management options taken on all seropositive cattle 2019-2022 as reported at RVIL, Nakuru |
|  | Five-year retrospective epidemiological assessment of Hydatidosis occurrence in Sheep and Goats, Igembe South, Meru County, 2021 | Retrospective Review of Brooding Chicks’ Records at Nakuru Regional Veterinary Investigation Laboratory for Avian Salmonellosis screening conducted, 2020-2021 | Assessing Human Dog Bites in Emgwen Sub County 2019-2021 |
|  | Quantification of causes of carcass and organ condemnation in cattle slaughtered at Kitengela abattoir, Kajiado County, Kenya, 2018 to 2019 | Retrospective study of Brucellosis cases at Garissa Regional Veterinary Investigation Laboratory, March 2021 to March 2022 | Knowledge, Attitude and Practices on Brucellosis among communities in Nandi County, Emgwen Sub County among Dairy Farmers July-September 2022 |
|  | Assessment of Economic Losses due to Organ condemnation in slaughtered Cattle in Ruiru Slaughterhouse, Kiambu County, January 2020 to August 2021 | Coliform Mastitis Prevalence and Antibiotic Susceptibility in Bovine Milk Samples Submitted at Regional Veterinary Investigation Laboratory (RVIL) Nakuru from January to April 2022 | Characterization of poultry diseases in Kericho County diagnosed at Regional Veterinary Investigation Laboratory, Kericho 2018-2021 |
|  | Prevalence and Economics of Hydatid cysts in camel (Camelus dromedaries) at Isiolo slaughter house, Burat ward, 2018 – 2020 | Knowledge, Attitude and Practices relating to Bovine Brucellosis disease among Farmers in Kurgung /Surungai Ward, Mosop – Nandi County | A Retrospective Data Review of the Prevalence and Spatial Distribution of Anthrax Cases in Livestock in Nakuru County, 2014-2021 |
|  | Mapping of Livestock routes to markets and Risk factors that catalyse the spread of Trans Boundary Diseases in Turkana County, July to August 2021 | Knowledge, Attitude and Practices of Livestock Farmers and Traders on Foot and Mouth Disease (FMD) in Rongo Sub County, Migori County, April –June 2022 | A retrospective Review on the Prevalence and Aetiology of Bovine Mastitis Diagnosed at Eldoret Regional Veterinary Investigation Laboratory, January 2020 to December 2022 |
|  | Assessment of Knowledge, Attitude and Practices of Indigenous Chicken Farmers to Poultry Biosecurity in Bumula Sub County-Bungoma County 2021 | Assessment of Knowledge, Attitude and Practices of Cattle Farmers on Foot and Mouth Disease among cattle farmers in Muhoroni Sub-County, Kisumu County, 2022 | Assessment of knowledge, attitude, and practices on Brucellosis among livestock farmers in Kapenguria Sub County, West Pokot County |
|  | Farmer perceptions on Newcastle disease in Mwingi west Sub County, Kitui County, Kenya | Assessment of Knowledge, Attitudes and Practices on Prevention and Control of Foot and Mouth Disease among Cattle Farmers in Thika Sub County, Kiambu County from April to June 2022 | Syndromic Analysis of Wildlife Clinical and Mortality Data in Kenya (April-June, 2022) |
|  | A Knowledge, Attitude and Practices of Coenurosis Infection in Sheep and Goats, Kajiado Central sub-County, July to August 2021 | Retrospective Study on Occurrence of Bovine Lumpy Skin Disease in Baringo County- Mogotio Sub-County, 2017-2021 | Knowledge, Attitude and Practices on Rabies among the residents of Maralal ward, Samburu Central Sub County, July -September 2022. |
|  | A Retrospective review of data to establish Vaccination Coverage and Trends and assess the Knowledge, Attitude and Practices towards Lumpy Skin Disease in Mathira | Knowledge, Attitude and Practices on East Coast Fever Immunization (ECFIM) amongst Livestock Keepers in North Kinangop Sub-County, 2022 | NYP |

*NYP= Not yet provided.*


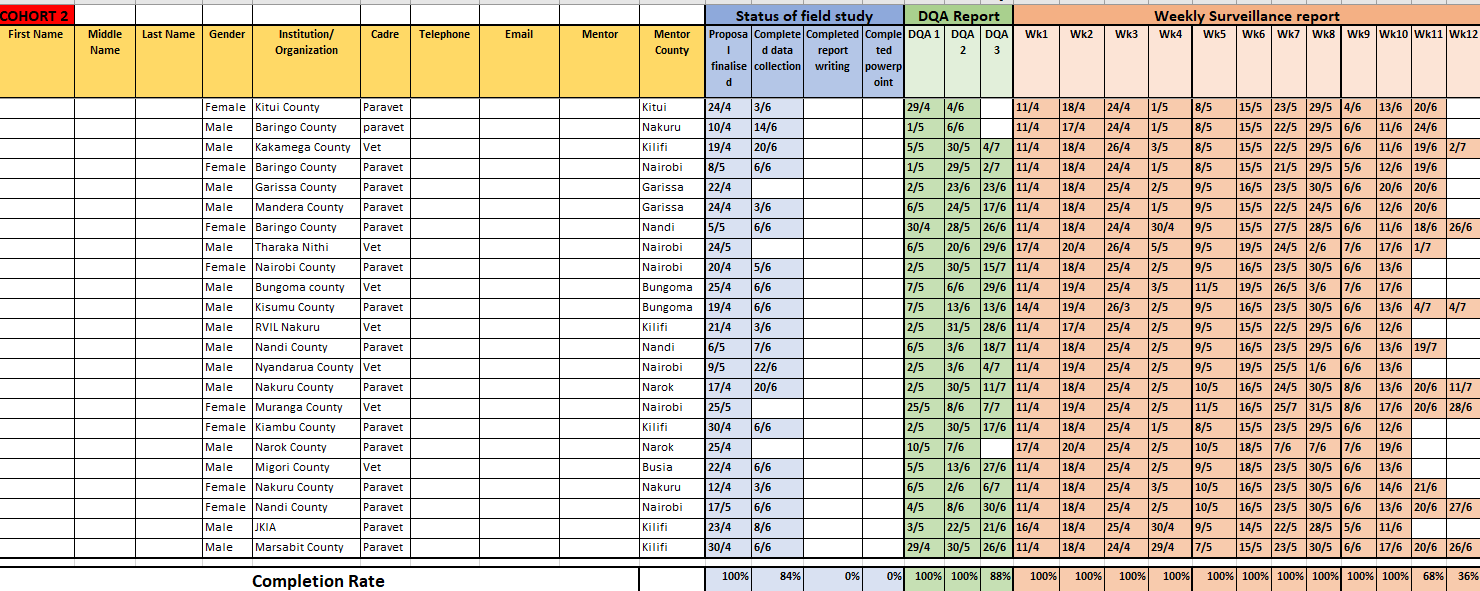


Supplementary Figure S2. Sample of the monitoring and evaluation dashboard to track the different component of the training and ensure compliance for the four-month long program. This tool is updated weekly until the completion of each cohort of trainees. The names and other identifiers were intentionally blanked out.
*DQA = data quality assessment, Wk =* week.

**Appendices: Questionnaires used in data collection**

**
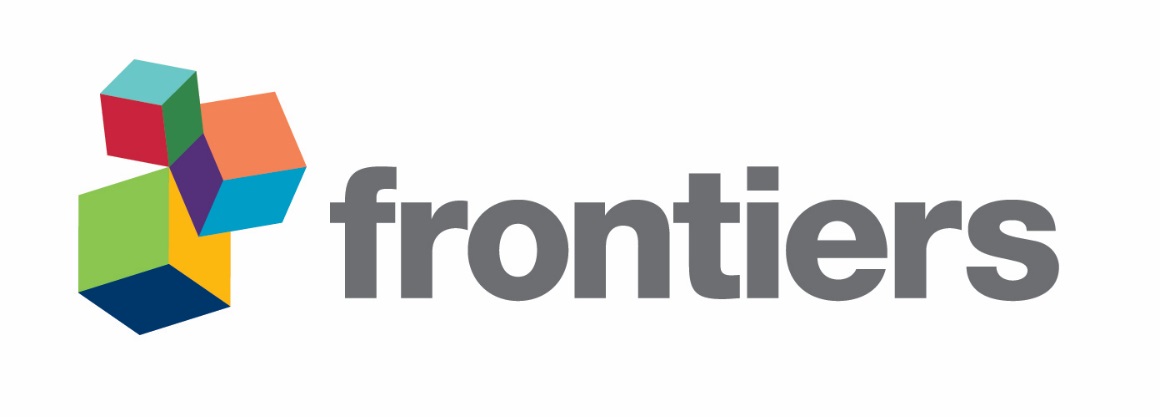
**
